# Supplementary material for: The autumnal lockdown was not the main initiator of the decrease in SARS-CoV-2 circulation in France
Source: Commun Med (Lond). 2021 Jun 30;1:7. doi: 10.1038/s43856-021-00002-6 (PMC9053270; doi:10.1038/s43856-021-00002-6)
Supplement: Supplementary file 1 — Supplementary Information [file 43856_2021_2_MOESM1_ESM.pdf]

Supplementary Figures

The autumnal lockdown was not the main initiator of the decrease in SARS-CoV-2 circulation in France

Authors: Veronica Pereda-Loth[1,2], Aldair Martínez Pineda[1], Lenka Tisseyre[1,2], Monique Courtade-Saidi[2], Christophe Bousquet[3], Camille Ferdenzi[3], Thierry Letellier[1], Moustafa Bensafi[3], Denis Pierron[1*]

Affiliations :
1- Équipe de Médecine Evolutive, Faculté de Chirurgie Dentaire, URU EVOLSAN Université Toulouse III, Toulouse, France
2- GSBMS, faculté de médecine Rangueil, Université Toulouse III, Toulouse, France
3- Lyon Neuroscience Research Center, CNRS UMR5292, INSERM U1028, Université Claude Bernard Lyon 1, Bron, France

*Correspondence: denis.pierron@univ-tlse3.fr

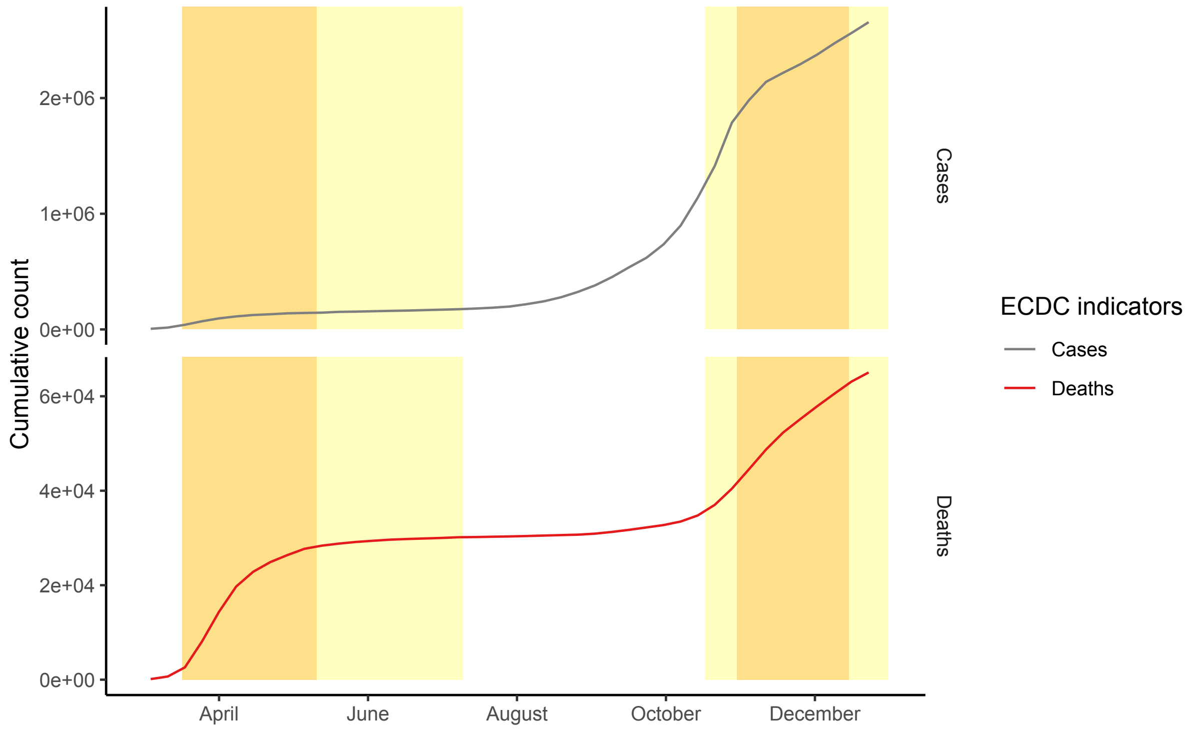

Supplementary Figure 1. Cumulative count of the number of case and Death associated to a COVID-19 infection in France during the year 2020. Orange rectangles represent periods of lockdown and yellow rectangle represent period of sanitary emergency state. Data are from the European Centre for Disease Prevention and Control (https://www.ecdc.europa.eu/en).

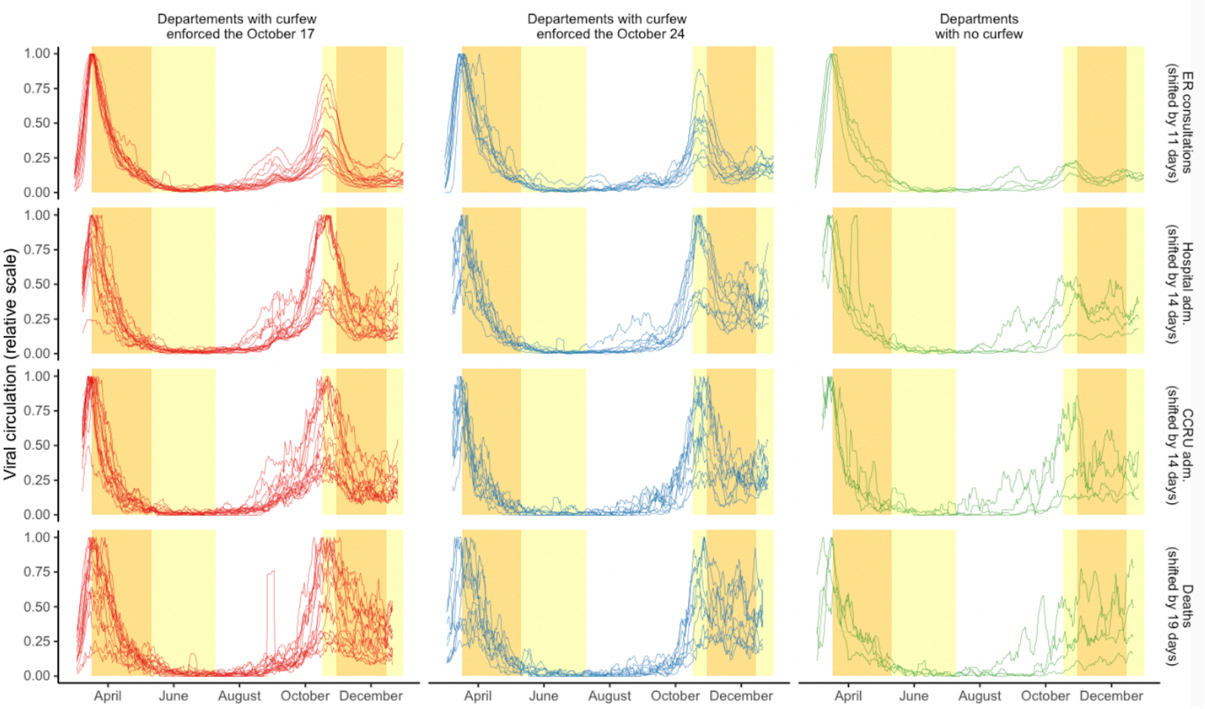

Supplementary Figure 2. Variation of the *estimation of the level of SARS-CoV-2 circulation in France in 2020 at the local scale. Each line represents one departement.* All the trends have been shifted by their respective number of days to get the peaks to align for the first peak. *For clarity only departements representing more than 1% of the COVID-19 hospitalization have been presented. Color of the lines represents the categorization of the departement during the autumnal peak. A) Phased governmental indicators: number of CCRU admissions, Hospital admissions, Number of daily deaths related to COVID and ER r*atio of consultations for suspected cases of COVID-19. ER= emergency room, CCRU= Critical care resuscitation unit, adm.= Admission.

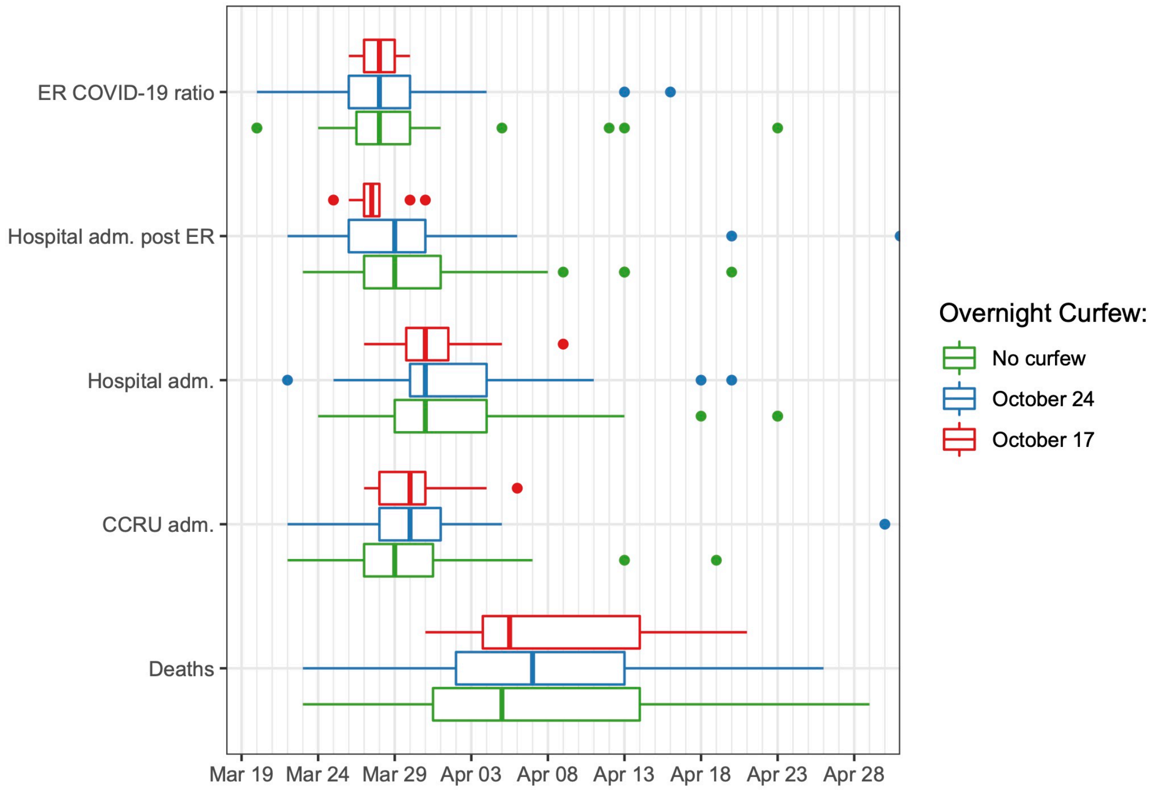

Supplementary Figure 3. Date of the maximum value (peak) of 7 epidemic indicators used to monitor SARS-CoV-2 circulation (unphased) in the departments during the spring lockdown 2020 according to the date of overnight curfew implementation of autumn 2020. Hospital admission post-ER represents the number of admissions to hospital after ER consultation. Box plots follow standard Tukey representations. French departments were assigned a red (n=16), blue (n=37) or green (n=43) label reflecting the date of implementation of overnight curfew enforcement. ER=emergency room, Adm.=Admission, CCRU= Critical care resuscitation unit admission.

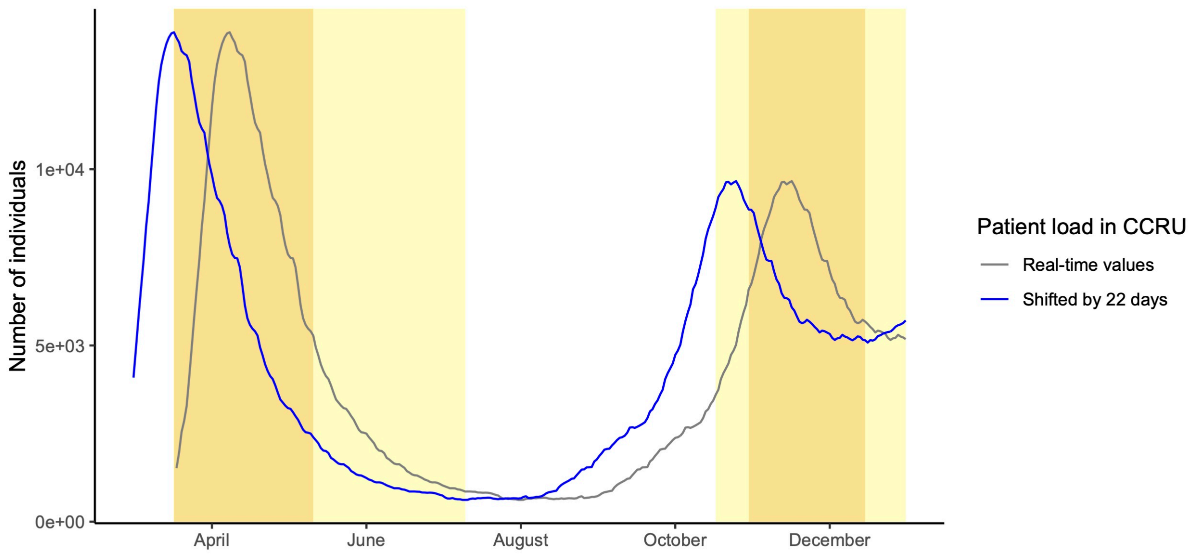

Supplementary Figure 4: Day to day number of COVID-19 patients in Critical Care Units (load) in France during the year 2020. Orange rectangles represent periods of lockdown and yellow rectangle represent period of sanitary emergency state. The blue line represents the real-time value reported by the French health system. The grey line represents the same data shifted by 22 days following our strategy to phase the peak with the peak of maximum of virus circulation the 17th march. CCRU= Critical care resuscitation unit admission.
