# Supplementary material for: The autumnal lockdown was not the main initiator of the decrease in SARS-CoV-2 circulation in France
Source: Commun Med (Lond). 2021 Jun 30;1:7. doi: 10.1038/s43856-021-00002-6 (PMC9053270; doi:10.1038/s43856-021-00002-6)
Supplement: Supplementary file 4 — Description of Additional Supplementary Files [file 43856_2021_2_MOESM4_ESM.pdf]

Description of Additional Supplementary Files

File Name: Supplementary Data 1

Description: Details of French governmental restrictions in Autumn.

File Name: Supplementary Data 2

Description: Raw data underlying all plots in the manuscript
