# Supplementary material for: The autumnal lockdown was not the main initiator of the decrease in SARS-CoV-2 circulation in France
Source: Commun Med (Lond). 2021 Jun 30;1:7. doi: 10.1038/s43856-021-00002-6 (PMC9053270; doi:10.1038/s43856-021-00002-6)
Supplement: Supplementary file 5 — Reporting Summary [file 43856_2021_2_MOESM5_ESM.pdf]

nature research

Corresponding author(s): Denis Pierron

Last updated by author(s): Apr 28, 2021

Reporting Summary

Nature Research wishes to improve the reproducibility of the work that we publish. This form provides structure for consistency and transparency in reporting. For further information on Nature Research policies, see our Editorial Policies and the Editorial Policy Checklist.

Statistics

For all statistical analyses, confirm that the following items are present in the figure legend, table legend, main text, or Methods section.

n/a	Confirmed	
☐	☒	The exact sample size (n) for each experimental group/condition, given as a discrete number and unit of measurement
☐	☒	A statement on whether measurements were taken from distinct samples or whether the same sample was measured repeatedly
☐	☒	The statistical test(s) used AND whether they are one- or two-sided<br>*Only common tests should be described solely by name; describe more complex techniques in the Methods section.*
☐	☒	A description of all covariates tested
☒	☐	A description of any assumptions or corrections, such as tests of normality and adjustment for multiple comparisons
☐	☒	A full description of the statistical parameters including central tendency (e.g. means) or other basic estimates (e.g. regression coefficient) AND variation (e.g. standard deviation) or associated estimates of uncertainty (e.g. confidence intervals)
☐	☒	For null hypothesis testing, the test statistic (e.g. F, t, r) with confidence intervals, effect sizes, degrees of freedom and P value noted<br>*Give P values as exact values whenever suitable.*
☒	☐	For Bayesian analysis, information on the choice of priors and Markov chain Monte Carlo settings
☒	☐	For hierarchical and complex designs, identification of the appropriate level for tests and full reporting of outcomes
☒	☐	Estimates of effect sizes (e.g. Cohen's d, Pearson's r), indicating how they were calculated

Our web collection on statistics for biologists contains articles on many of the points above.

Software and code

Policy information about availability of computer code

Data collection	https://form.crnl.fr/index.php/146862?newtest=Y&lang=fr , https://www.gouvernement.fr/info-coronavirus, https://trends.google.com/,https://geodes.santepubliquefrance.fr/#c=home.
Data analysis	R 4.0.0 GUI 1.71 Catalina build (7827)<br>Code for data cleaning and analysis associated with this manuscript is available at: https://github.com/DenisPierron/COVID-19/blob/main/codeCOM.zip

For manuscripts utilizing custom algorithms or software that are central to the research but not yet described in published literature, software must be made available to editors and reviewers. We strongly encourage code deposition in a community repository (e.g. GitHub). See the Nature Research guidelines for submitting code & software for further information.

Data

Policy information about availability of data

All manuscripts must include a data availability statement. This statement should provide the following information, where applicable:

- Accession codes, unique identifiers, or web links for publicly available datasets
- A list of figures that have associated raw data
- A description of any restrictions on data availability

All data underlying all plots in the manuscript is available on the Supplementary Data 2.

Field-specific reporting

Please select the one below that is the best fit for your research. If you are not sure, read the appropriate sections before making your selection.

☐ Life sciences ☒ Behavioural & social sciences ☐ Ecological, evolutionary & environmental sciences

For a reference copy of the document with all sections, see nature.com/documents/nr-reporting-summary-flat.pdf

Behavioural & social sciences study design

All studies must disclose on these points even when the disclosure is negative.

Study description	this study attempt to follow the level of circulation of SARS-CoV-2 in France in autumn 2020.<br>It focus on the effect of measures such as curfew and lockdown.<br>Study type/design: Observational study, Quantitative cross-sectional
Research sample	Participants were recruited by word of mouth as well as through social and traditional media (flyers, social media, television, radio) during the COVID-19 pandemic. It was well covered by the French press, at both the regional and national level. Respondents received no monetary incentive for their participation.
Sampling strategy	No sampling strategy was applied to this study.
Data collection	Governmental indicators regarding the healthcare system in France (ER consultation, hospitalizations, CCRU admission and deaths, PCR) were downloaded on February 25th 2021 from the French Public Health website https://geodes.santepubliquefrance.fr/#c=home. Hospitalization, CCRU admission and deaths indicators correspond to the new number of cases by day. ER consultation ratio corresponds to the ratio of consultation with medical diagnosis of COVID-19 over the general consultations at the emergency room (ER) in hospitals. Number of detected new viral infections (positive and negative) based on nucleic acid are also downloaded from the French Public Health website. We report the raw number as well as the rate of daily positive diagnosis.<br>Online survey was conducted in the French population between April 8 and January 10, 2021 and aimed at characterizing chemosensory disorders in people with and without COVID-19, as well as their consequences on quality of life. 4628 responses were analyzed (Supplementary Data 2). This survey was approved by the CNRS ethics committee. Data collection was strictly anonymous. The protocol complies with the revised Declaration of Helsinki and the study was approved by the ethics committee of the Institute of Biological Sciences of the CNRS on the 3rd of April 2020 (DPO #TRRECH-467). All individuals provided informed consent when participating in the survey.<br><br>The study of online queries was downloaded from Google Trends (https://trends.google.com/), using the R library gtrendsR. We looked separately for the popularity of the terms: loss of smell (perte odorat) and loss of taste (perte goût), using default selection of All categories and Web search), within the timeframe of March 1st, 2020 to January 10th, 2021. Google Trends do not provide the actual numbers of searches but rather a relative score from 0 to 100 (100 corresponding to the day with the greatest number of searches during the specified time period).
Timing	Online survey was conducted in the French population between April 8 2020 and January 10th 2021<br>For each indicator we report the available data between 1st march 2020 to January 10th 2021.
Data exclusions	no data were excluded on google and healthcare system.<br>For local questionnaire Inclusion criteria were as follows. i) Questionnaire completion was allowed only to participants who indicated they had suffered from a respiratory disease in the past two weeks, whether they noticed a change in their taste/smell or not. ii) Participants aged 18 years old or younger were excluded.
Non-participation	NA
Randomization	This is an observational study.

Reporting for specific materials, systems and methods

We require information from authors about some types of materials, experimental systems and methods used in many studies. Here, indicate whether each material, system or method listed is relevant to your study. If you are not sure if a list item applies to your research, read the appropriate section before selecting a response.

Materials & experimental systems

n/a	Involved in the study	
☒	☐	Antibodies
☒	☐	Eukaryotic cell lines
☒	☐	Palaeontology and archaeology
☒	☐	Animals and other organisms
☐	☒	Human research participants
☒	☐	Clinical data
☒	☐	Dual use research of concern

Methods

n/a	Involved in the study	
☒	☐	ChIP-seq
☒	☐	Flow cytometry
☒	☐	MRI-based neuroimaging

Human research participants

Policy information about studies involving human research participants

Population characteristics	see above
Recruitment	Participants were recruited by word of mouth as well as through social and traditional media (flyers, social media, television, radio) during the COVID-19 pandemic. As many COVID-19 surveys, including our own, suffer from biases in the profile of respondents, as they may be more willing and interested in participating in scientific research.
Ethics oversight	The protocol complies with the revised Declaration of Helsinki and the study was approved by the ethics committee of the Institute of Biological Sciences of the CNRS on the 3rd of April 2020 (DPO #TRRECH-467). All individuals provided informed consent when participating in the survey.

Note that full information on the approval of the study protocol must also be provided in the manuscript.
